# Maternal postpartum bonding impairment and increased substance use to cope with pandemic-related stress

**DOI:** 10.3389/fpsyg.2024.1275857

**Published:** 2024-04-17

**Authors:** Alysa Roland, Caitlin M. Dressler, Karina M. Shreffler

**Affiliations:** Fran and Earl Ziegler College of Nursing, University of Oklahoma Health Sciences Center, Oklahoma City, OK, United States

**Keywords:** maternal, substance use, COVID-19 pandemic, Postpartum Bonding Questionnaire (PBQ), coping, mental health

## Abstract

Substance use rates, particularly among women, increased substantially during the COVID-19 pandemic. Psychological and economic risks disproportionately experienced by women were associated with increase in substance use patterns during the pandemic. Using substances (i.e., tobacco, alcohol, cannabis) to cope with stress is well-documented; what is less known are protective factors that were associated with less substance use during the pandemic. We examined whether mothers of young children (*N* = 96) who reported postpartum bonding impairment before the start of the pandemic reported greater substance use during the pandemic as a means to cope. Results from linear regression analyses found that mothers who reported higher levels of pre-pandemic mother-infant bonding impairments reported greater use of alcohol and other substances as a means to cope with pandemic stressors. These findings suggest that social connections might be a strategy to reduce substance use as a maladaptive coping behavior, especially during widespread crises such as the recent pandemic or for mothers of young children. In particular, promoting postpartum bonding through interventions might help to reduce substance use among new mothers.

## Introduction

In the decade leading up to the global COVID-19 pandemic, rates of heavy and binge drinking among U.S. women increased by more than 50% (Grant et al., 2017). Substance abuse, particularly among women, tends to increase during or following crisis (Cepeda et al., 2010; Mulia et al., 2014), often in response to stressful conditions as a maladaptive coping mechanism (Abbey et al., 1994). Beyond the direct health impacts of the virus, the global COVID-19 pandemic brought unprecedented economic and social stressors that disproportionately affected women in the U.S. (Burki, 2020; Connor et al., 2020). Among all adults, substance use—including tobacco, alcohol, and cannabis—increased during the pandemic (Bommelé et al., 2020; Chong et al., 2022; Sohi et al., 2022). Women’s alcohol consumption in the U.S. increased by 41% in 2020 compared to rates 1 year earlier (Pollard et al., 2020). A review of studies on substance use behaviors during the pandemic found that mental health disorders, particularly depression, as well as anxiety, hopelessness, and social isolation were associated with increased use of alcohol and other substances (Roberts et al., 2021).

Maternal substance abuse is a substantial public health concern due to its numerous adverse implications for maternal and child health and well-being. Substance abuse among mothers of young children can increase the risk for child maltreatment (Young et al., 2007) and is the most common reason for referral to child protective services (Parolin and Simonelli, 2016). When compared to mothers who do not abuse substances, mothers with substance use disorders are more likely to have mother–child interaction problems (Punamäki et al., 2021) or to meet their children’s emotional and physical needs (Bauman and Dougherty, 1983; Mayes and Truman, 2002). Due to the increase in women’s substance use leading up to and during the pandemic, it is critical to identify factors that were protective against increased substance use among mothers of young children.

Social support and connectedness during the pandemic have been identified as protective factors against pandemic stressors (e.g., Shreffler et al., 2021; Zhou et al., 2021). Typically, impacts of social connectedness are examined within one’s social network (Nitschke et al., 2021) or intimate partnerships (Lee and Robbins, 1995). Yet parent–child connectedness is also associated with well-being outcomes among both parents and children (An and Cooney, 2006; Boutelle et al., 2009). The early caregiver-infant bonding relationship has been identified as a key motivation in preventing or reducing substance use before the pandemic (Sanjuan et al., 2019). There is likely a complex relationship between maternal mental health, maternal–infant bonding, and substance use in early motherhood. Women with greater depressive symptoms are more likely to report maternal–infant bonding impairment (Kerstis et al., 2016), which suggests that women with greater depressive symptoms and impaired bonding would higher reported use of substances to cope with pandemic-related stressors. The purpose of this study is to examine whether pre-pandemic mother-infant bonding measured at 6 months postpartum served as a protective factor for using substances to cope with COVID-19 pandemic stressors among a diverse and low-income sample of mothers of young children, after adjusting for sociodemographic characteristics including age, race/ethnicity, educational attainment, and union status, as well as postpartum depressive symptoms. We hypothesize that participants who reported postpartum bonding impairment before the pandemic would report increased substance use for coping during the pandemic.

## Methods

### Participants

Data for this study comes from a longitudinal sample of low-income and racially and ethnically diverse mothers (aged 16–38) recruited in 2017–2018 in the South Central U.S. during their first prenatal visit. Participants were eligible if they were planning to continue their pregnancy and be a primary caregiver to their offspring, and they had to be less than 16 weeks gestation at their first assessment (average gestation was 10 weeks). Participants were ineligible if they did not speak and read English. The authors’ university Institutional Review Board approved all study procedures, and all participants provided written consent or assent with parental consent for participants under 18. The sample for the current study (*N* = 96) includes those who participated through the last survey assessment conducted in November–December 2020 and answered all questions included in this study.

### Procedure

**Figure 1:**
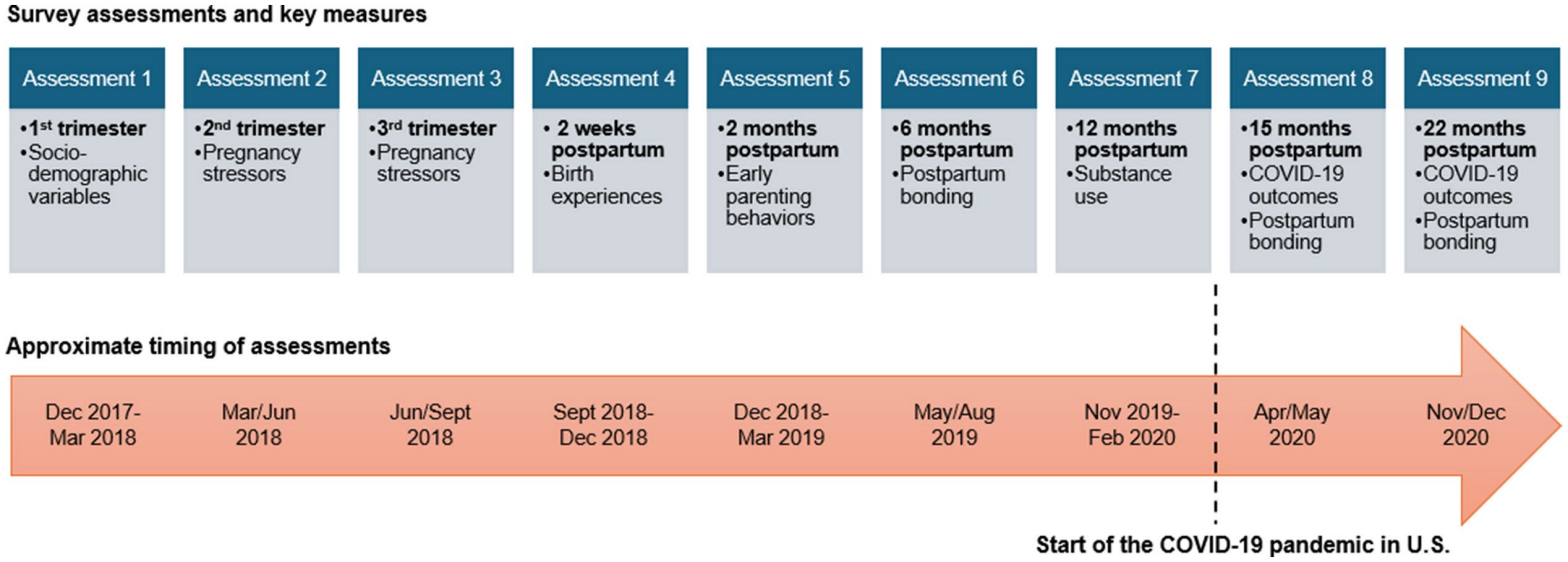
Timing and key measures of survey assessments.

Nurses at the first prenatal visit screened potential participants for eligibility and introduced the study. Those who were interested in participating met with members of the research team following their prenatal appointment. At that time, the research team members reviewed the study procedures and collected written informed consent or parental consent and assent for those under 18, and they reviewed the compensation information with participants. Once participants were enrolled in the study, all assessments were conducted online using REDCap survey software. Participants were compensated between $25–50 for completing each survey assessment (amounts differed due to length of the survey and estimated completion time). There were three survey assessments conducted during pregnancy and six assessments conducted post-birth with an overall goal of understanding mechanisms linking maternal stressors to postpartum maternal and child health and well-being outcomes (see Figure 1 for a timeline of data collection and key constructs). Assessments 1–7 were conducted prior to the pandemic and Assessments 8 and 9 were conducted during the first year of the COVID-19 pandemic (in April/May 2020 and November/December 2020, respectively).

### Measures

Sociodemographic characteristics were assessed in the first survey assessment. Maternal age was measured in years. Race/ethnicity was coded into dummy variables using Census coding guidelines and includes Black, Hispanic, and Native American with non-Hispanic White as the reference category. Education measured in years was used as a measure of socioeconomic status. Union status (married or cohabiting = 1/single = 0) was included as a dichotomous variable. Depressive symptoms were assessed in the fifth assessment, occurring approximately 2 months postpartum, using the Center for Epidemiologic Studies Depression (CESD) scale (Radloff, 1977).

The Postpartum Bonding Questionnaire (PBQ; Brockington et al., 2001) was assessed at the six-month post-birth survey. The PBQ is a 24-item scale coded/recoded from 0 to 5 so that higher values indicate bonding impairment. The range for the PBQ scale in this sample is 0–73, and the Cronbach’s alpha reliability score for this sample is 0.91. The mean for bonding impairment is 6.17 in this sample. Prior research validating the PBQ has identified a score of 25 as indicating a bonding disorder, with a score of 40 indicating severe bonding disruption (Brockington et al., 2006).

Questions assessing participants’ usage of substances to cope with pandemic stressors were asked in Assessment 8 (April/May 2020) and 9 (November/December 2020). Participants were provided the following prompt regarding how they were coping with the pandemic:

“These items deal with ways you have been coping with the stress in your life related to the coronavirus. There are many ways to try to deal with problems. These items ask what you have been doing to cope with this one. Obviously, different people deal with things in different ways, but I’m interested in how you have tried to deal with it. Each item says something about a particular way of coping. We want to know to what extent you have been doing what the item says. How much or how frequently. Do not answer on the basis of whether it seems to be working or not.”

Items related to substance use for coping included: “I’ve been using alcohol or other drugs to make myself feel better” and “I’ve been using alcohol or other drugs to get through it.” Response options ranged from 0 = “I have not done this at all,” to 3 = “I’ve been doing this a lot” for each question across the two post-pandemic survey waves and then summed to create an index score ranging from 0 to 12.

### Analytical strategy

For descriptive purposes, we examined the means and standard deviations of study variables. Multiple linear regression analysis was used to examine the association between pre-pandemic postpartum bonding impairment scores and self-reported increases in alcohol and other substances use to cope with COVID-19-related stressors, controlling for sociodemographic characteristics and depressive symptoms. We ran several sensitivity analyses to examine whether results changed depending upon the timing of the depression assessment, including prenatal depressive symptoms (measured in the 3rd trimester), postpartum depressive symptoms (measured at 2 months postpartum), and postpartum depressive symptoms (measured at about 1.5 years postpartum, after the start of the COVID-19 pandemic).

## Results

Descriptive statistics are presented in Table 1. The average coping-related substance use increase score was 1.18 (SD = 2.49), showing a mild increase in use in terms of the index scale range (0–12). The average age of the sample was 26.19 (SD = 5.65). The sample was racially and ethnically diverse, including White (40%), Black (31%), Hispanic (12%), and Native American (17%) women. The average educational attainment was 13.07 years (SD = 2.06). The majority of participants reported their union status as being married or cohabiting (58%). The average depression score assessed at 2 months postpartum was 12.48 (SD = 8.63). Postpartum bonding impairment was low among the women in the sample, with an average score of 6.17 (SD = 10.80).

**Table 1:** Descriptive statistics of study variables (*N* = 96).

Variables	M	SD
Coping-related increase in substance use	1.18	2.49
Age	26.19	5.65
White	0.40	0.49
Black	0.31	0.46
Hispanic	0.12	0.33
Native American	0.17	0.37
Education in years	13.07	2.06
In union (married/cohabiting)	0.58	0.50
Depressive symptoms (2 mo PP)	12.48	8.63
Postpartum bonding impairment (6 mo PP)	6.17	10.80

PP, Postpartum.

Unstandardized b coefficients, standard errors, standardized beta coefficients, *p*-values, and confidence intervals at the 95% level for the multiple regression analysis results are presented in Table 2. The first model included sociodemographic control variables and depressive symptoms measured at 2 months postpartum and found no significant results. The second model included impaired bonding, assessed at 6 months postpartum (approximately 9 months before the start of the COVID-19 pandemic in the U.S.). We found that mothers who reported greater postpartum bonding impairment *before* the pandemic reported greater use of alcohol or other drugs to cope with pandemic-related stressors (*b* = 0.08; *p* < 0.05), controlling for sociodemographic characteristics such as age, race/ethnicity, educational attainment, and union status, as well as for postpartum depressive symptoms. Additional tests for sensitivity utilizing measures of prenatal depressive symptoms or postnatal depressive symptoms assessed after the start of the COVID-19 pandemic did not reveal new information.

**Table 2:** Multiple linear regression analysis of postpartum bonding impairment and self-reported use of alcohol and other substances to cope with COVID-19 pandemic stressors (*n* = 96).

	Model 1					Model 2				
*Variables*	*b*	*SE*	*Beta*	*p-value*	*CI*	*b*	*SE*	*Beta*	*p-value*	*CI*
***Sociodemographic controls***										
Age	−0.10	0.06	−0.23	0.09	[−0.21 to 0.02]	−0.14	0.06	−0.34	0.02	[−0.25 to −0.03]
White (reference)										
Black	0.58	0.68	0.12	0.40	[−0.79 to 1.95]	0.22	0.67	0.04	0.75	[−1.13 to 1.57]
Hispanic	−1.28	0.92	−0.19	0.17	[−3.12 to 0.55]	−1.53	0.89	−0.23	0.09	[−3.30 to 0.25]
Native American	−1.03	0.86	−0.15	0.24	[−2.74 to 0.69]	−0.90	0.83	−0.13	0.28	[−2.55 to 0.75]
Education	0.03	0.15	0.02	0.86	[−0.28 to 0.34]	0.07	0.15	0.06	0.64	[−0.23 to 0.37]
Union	−0.45	0.63	−0.09	0.48	[−1.72 to 0.82]	−0.82	0.63	−0.17	0.20	[−2.07 to 0.44]
Depressive symptoms (2 mo PP)	0.04	0.03	0.16	0.22	[−0.03 to 0.11]	0.02	0.03	0.08	0.52	[−0.05 to 0.09]
PP bonding impairment (6 mo PP)						0.08	0.03	0.31	0.02	[0.01 to 0.14]
Constant	2.98	2.38		0.22	[−1.78 to 7.74]	3.80	2.31		0.11	[−0.83 to 8.43]
Adjusted R^2^	0.05					0.12				

^***^p < 0.001; ^**^p < 0.01; ^*^p < 0.05. PP, Postpartum.

## Discussion

Our study examined associations between mother-infant bonding measured at 6 months postpartum (measured in summer of 2019) and self-reported use of alcohol or other substances in order to cope with the pandemic (measured in April/May 2020 and November/December 2020). Our inferential findings showed that mothers of young children who reported higher levels of bonding impairment before the pandemic reported greater use of alcohol or other drugs to make themselves feel better due to the pandemic.

The importance of the postpartum bonding relationship is critical for maternal well-being, the health and development of the child, and the mother-infant relationship (Brockington, 2004; Lutkiewicz et al., 2020). This bond might be especially important during times of widespread societal stressors such as the pandemic, where the lack of social support due to physical distancing and quarantine might have played a role in mothers’ desire to use substances postpartum due to the adjustments of being a parent. These changes in the normal pattern of socialization and routine have been identified as the more prominent stressors of the pandemic for families (Adams et al., 2021; Jones et al., 2022). Under these circumstances, mothers are adjusting to a new or different role and are simultaneously coping with the social limitations of the pandemic. Maternal postpartum depression has been linked to substance use (Slomian et al., 2019) and increased bonding impairment at 6 months postpartum (Kerstis et al., 2016), but we did not find a significant association between depressive symptoms and self-reported increased substance use during the pandemic.

These findings suggest that close social connectedness is a protective mechanism for mothers with young children against substance use. Future research is needed to examine this association. Previous research has identified factors that affect one’s ability to bond, and those same factors might affect substance use behaviors. A mother’s psychological well-being, for example, has implications for whether or not a successful mother-infant bond will be able to develop (Liu et al., 2022; Doyle et al., 2023). Similarly, prior research has found that mothers who had greater well-being prior to the pandemic displayed more effective parenting behaviors and emotion regulation skills during the pandemic (Jones et al., 2022). Although we did not find that maternal depressive symptoms were associated with an increase in substance use during the pandemic, it is possible that those who were more depressed did not recognize an increase in their substance use behaviors. Objective measurement of substance use as opposed to self-reports might have more validity.

The data and methods of this study had several limitations, however. The sample was small and geographically limited to women receiving prenatal care at two prenatal clinics in an urban city. As pandemic policies differed widely across states and metropolitan areas, we cannot extrapolate findings to other settings or among different samples. Measures were obtained through self-assessments of maternal bonding and the use of alcohol and other substances to cope with pandemic-related stressors, which might be subject to recall bias or impacted by social desirability bias. Overall, impaired bonding rates in our sample were low, limiting the possibility of examining subscale factors of the PBQ such as “risk of abuse” on substance use outcomes. Moreover, measures on substance use were collected via self-report which is a less reliable measure than toxicology testing (Lendoiro et al., 2013; Young-Wolff et al., 2020), especially in the perinatal population, who may fear the legal consequences of disclosure. Harm reduction approaches are needed to encourage women to disclose and seek treatment for substance use (Van Scoyoc et al., 2017). Lastly, it is possible that there might be unmeasured confounders that are associated with bonding and substance use for coping scores. Our decision to include maternal depressive symptoms measured at 2 months postpartum (before the measurement of bonding and before the onset of the COVID-19 pandemic) were an attempt to control for maternal mental health, as prior research suggests depression is associated with impaired bonding and greater substance use behaviors. We also ran sensitivity analyses to assess whether depressive symptoms assessed during pregnancy or during the pandemic might influence results differently than those assessed 2 months postpartum and did not find any differences. Still, it is possible that other unmeasured factors such as economic stressors, attachment styles, personality characteristics, or personality disorders might affect both bonding and maladaptive coping mechanisms such as substance use.

Despite limitations, this study had important strengths as well. Notably, the longitudinal nature of the study allowed for the examination of pre-pandemic factors on outcomes during the pandemic in an unprecedented natural experiment. The sample was diverse and predominantly low-income but not designed to be a sample of substance-abusing mothers or a sample of mothers with bonding impairments.

## Conclusion

Findings of this study highlight the relationship between pre-pandemic postpartum bonding impairment and increased substance use to cope with pandemic stressors among mothers of young children. These findings have critical implications for clinicians and researchers because they suggest that mothers with more impaired bonding might be at risk of using substances to cope during a pandemic or times of stress and point to the need for intervention such as parenting education and enhanced bonding as a way to promote effective coping and relationship skills. Although the purpose of this study was not to examine the impact of maternal mental health on postpartum bonding impairments, the association between maternal mental health and maternal–infant bonding is well-known. Identifying depression in the early postpartum period might help to reduce bonding impairment later in the postpartum period and also provide an opportunity to for clinicians to work with new mothers on positive coping behaviors to reduce the need to use substances to cope with stress. Clinicians caring for postpartum mothers with depression should also screen for bonding impairments and stress the importance of social connection to their patients. This study suggests that parenting education interventions designed to enhance bonding have the potential to reduce maternal alcohol and other substance use as coping behaviors. Future studies can examine the effectiveness of interventions designed to enhance postpartum bonding on substance use behaviors, especially in low-income and racially and ethnically diverse mothers.

## Data availability statement

The raw data supporting the conclusions of this article will be made available by the authors, without undue reservation.

## Ethics statement

The studies involving humans were approved by Oklahoma State University Institutional Review Board. The studies were conducted in accordance with the local legislation and institutional requirements. Written informed consent for participation in this study was provided by the participants’ legal guardians/next of kin.

## Author contributions

AR: Writing – original draft. CD: Writing – original draft. KS: Conceptualization, Data curation, Formal analysis, Methodology, Supervision, Writing – review & editing.
